# Clinical Significance of Pregnancies Complicated by Velamentous Umbilical Cord Insertion Associated With Other Umbilical Cord/Placental Abnormalities

**DOI:** 10.14740/jocmr2310w

**Published:** 2015-09-25

**Authors:** Shunji Suzuki, Masahiko Kato

**Affiliations:** a Department of Obstetrics and Gynecology, Japanese Red Cross Katsushika Maternity Hospital, Tokyo, Japan

**Keywords:** Velamentous umbilical cord insertion, Adverse outcome, Placental abnormalities

## Abstract

**Background:**

We examined the clinical significance of pregnancies complicated by velamentous cord insertion (VCI) associated with other umbilical cord/placental abnormalities.

**Methods:**

Data were collected from 168 deliveries complicated by VCI and from 16,797 unaffected controls. All placentae were screened identically by trained staff. In this study, we examined the presence of excessively long umbilical cord (longer than 70 cm), vasa previa, single umbilical artery, circumvallate placenta, succenturiate placenta, lobed placenta, placenta previa, low lying placenta and placenta accrete as the other umbilical cord/placental abnormalities.

**Results:**

Using a multivariate analysis, the pregnancies complicated by VCI were independently associated with *in vitro* fertilization use (P < 0.01), maternal smoking (P = 0.03), preterm delivery (P = 0.03), fetal asphyxia (P = 0.01) and small-for-gestational-age infants (P = 0.02). It was also independently associated with vasa previa (P < 0.01), single umbilical artery (P = 0.04), lobed placenta (P = 0.01) and placenta previa (P = 0.03). However, these umbilical cord/placental abnormalities were not associated with the further adverse outcomes of the pregnancies complicated by VCI.

**Conclusion:**

VCI is associated with an increased risk of adverse perinatal outcomes irrespective of the presence of other umbilical cord/placental abnormalities. Routine identification of the placental cord insertion site should be considered.

## Introduction

Velamentous cord insertion (VCI) is a defect in the insertion site of the umbilical cord resulting from the atrophy of portions of the developing placenta. This condition has been reported to be associated with preterm labor-delivery, low birth weight, fetal growth restriction, abnormal intrapartum fetal heart rate patterns, low Apgar scores at 1 and 5 min, neonatal deaths and placental abruption [1, 2]. VCI is a defect in the insertion site of the umbilical cord resulting from the atrophy of portions of the developing placenta. In this condition, the blood vessels of the umbilical cord are not protected by Wharton’s jelly, resulting in a potential for increased risk of breakage when the amniotic membranes rupture [3]. Abnormal cord insertions such as VCI have been supposed to develop secondary to an oblique orientation of the blastocyst at nidation as one of some hypotheses [4-6]. If the embryo does not face the base of implantation, the umbilical vessels must extend between the connecting stalk and the placenta in order to reach the endometrium, where the placenta can develop appropriately, thus resulting in either marginal or membranous insertion. To date, therefore, VCI has been supposed to be associated with the high incidence of other umbilical cord/placental abnormalities such as vasa previa, placenta previa, low lying placenta and placenta accrete associated with the adverse perinatal outcomes [4, 7-11]. However, there might have not been any investigations those considered whether or not the prognosis of pregnancies complicated by VCI is made worse by other umbilical cord/placental abnormalities. In this study, therefore, we examined the clinical significance of pregnancies complicated by VCI associated with other umbilical cord/placental abnormalities.

## Methods

The material reviewed consisted of the total population of women who gave birth to singleton babies at Japanese Red Cross Katsushika Maternity Hospital between 2002 and 2011. Demographic information and the characteristics of labor were extracted from patient charts. Data were collected from 168 deliveries complicated by VCI and from 16,797 unaffected controls. Our departmental protocol does not dictate that the cord insertion site should be evaluated. All placentae were screened identically by trained staff. In this study, we examined the presence of excessively long umbilical cord (longer than 70 cm), vasa previa, single umbilical artery, circumvallate placenta, succenturiate placenta, lobed placenta, placenta previa, low lying placenta and placenta accrete as the other umbilical cord/placental abnormalities. The diagnosis of umbilical cord/placental abnormalities was made macroscopically and confirmed by placental pathology. The gestational age of the pregnancies were established by ultrasonographic examination of the fetal crown-rump length at 8 - 11 weeks’ gestation. Small-for-gestational-age (SGA) infants were defined as those with sex- and aged-adjusted birth heights and weights below and over the 10th percentile according to the fetal growth curve for Japanese (Acta Neonatol Jpn from Japan Society of Neonatology). The fetus was considered to be in “asphyxia” if repeated late or severe variable deceleration (< 60 beats/min and ≥ 60 s) and/or prolonged decelerations (< 100 beats/minute and ≥ 5 min) occurred. Neonatal asphyxia was defined by an Apgar score < 7 at 5 min and/or umbilical arterial pH < 7.0. Placental abruption was defined as complete or partial separation of a normally implanted placenta occurring before delivery, confirmed at delivery by evidence of retro-placental bleeding at delivery and placental pathology. Hypertensive disorders were defined as blood pressure > 140/90 mm Hg measured on two or more occasions at least 6 h apart with the patient under bed rest.

The following clinical characteristics and outcomes were analyzed: parity; maternal age; *in vitro* fertilization (IVF) use; maternal smoking; maternal hypertensive disorders; placental abruption; gestational age; birth weight; intrauterine fetal demise; delivery modes; fetal and neonatal asphyxia.

Data are presented as number (%). For statistical analysis of the categorical variables, the Χ^2^ test with Yates’ correction was used. Differences with P < 0.05 were considered significant. Odds ratios and 95% confidence intervals (CIs) were also calculated. Variables used in the multivariate model were those that on univariate analysis had shown statistical significance toward association with increased risk of the adverse outcomes.

## Results

During the study period, there were 16,965 singleton deliveries in our hospital. Of these, 168 (1.0%) were complicated by VCI.

Table 1 shows clinical characteristics and outcomes of the singleton pregnancies with and without VCI. The pregnancies complicated by VCI were more likely to be associated with nulliparity, IVF use, maternal smoking, hypertensive disorders, placental abruption, preterm delivery, emergency cesarean delivery, fetal asphyxia, neonatal asphyxia and SGA infants as compared to those without VCI. Using a multivariate analysis, the pregnancies complicated by VCI were independently associated with IVF use (adjusted OR: 2.01; 95% CI: 1.2 - 3.3; P < 0.01), maternal smoking (adjusted OR: 1.43; 95% CI: 1.0 - 2.3; P = 0.03), preterm delivery (adjusted OR: 1.74; 95% CI: 1.1 - 2.8; P = 0.03), fetal asphyxia (adjusted OR: 1.47; 95% CI: 1.0 - 2.6; P = 0.01) and SGA infants (adjusted OR: 1.68; 95% CI: 1.1 - 2.9; P = 0.02).

**Table 1:** Clinical Characteristics and Outcomes of the Singleton Pregnancies With and Without Velamentous Umbilical Cord Insertion

Velamentous cord insertion	No	Yes	P-value	Odds ratio	95% CI
Total	16,797	168			
Maternal age ≥ 35 years	5,755 (34.3)	63 (37.5)	0.37	-	-
Nulliparity	8,655 (51.5)	114 (6.8)	< 0.01	1.99	1.4 - 2.8
*In vitro* fertilization use	900 (5.4)	36 (21.4)	< 0.01	4.82	3.3 - 7.0
Maternal smoking	357 (2.1)	9 (5.4)	< 0.01	2.61	1.3 - 5.1
Hypertensive disorders	702 (4.2)	18 (10.7)	< 0.01	2.75	1.7 - 4.5
Placental abruption	101 (0.6)	5 (3.0)	< 0.01	5.07	2.0 - 13
Intrauterine fetal demise	124 (0.7)	3 (1.8)	0.11	-	-
Preterm delivery	1,349 (8.0)	37 (22.0)	< 0.01	3.23	2.2 - 4.7
Emergency cesarean delivery	1,439 (8.6)	29 (17.3)	< 0.01	2.23	1.5 - 3.3
Fetal asphyxia	993 (5.9)	20 (11.9)	< 0.01	2.15	1.3 - 3.5
Neonatal asphyxia	7 (4.2)	240 (1.4)	< 0.01	3.00	1.4 - 6.5
Small-for-gestational-age infant	936 (5.6)	22 (13.1)	< 0.01	2.55	1.6 - 4.0

Data are presented as number (%). CI: confidence interval.

Table 2 shows the relation between VCI and other umbilical cord/placental abnormalities. The pregnancies complicated by VCI were more likely to be associated with vasa previa, single umbilical artery, circumvallate placenta, lobed placenta, placenta previa and placenta accrete. Using a multivariate analysis, it was also independently associated with vasa previa (P < 0.01), single umbilical artery (adjusted OR: 2.01; 95% CI: 0.92 - 16; P = 0.04), lobed placenta (adjusted OR: 4.70; 95% CI: 1.0 - 18; P = 0.01) and placenta previa (adjusted OR: 3.25; 95% CI: 0.91 - 5.5; P = 0.03).

**Table 2:** Relation Between Velamentous Umbilical Cord Insertion and Other Umbilical Cord/Placental Abnormalities

Velamentous cord insertion	No	Yes	P-value	Odds ratio	95% CI
Total	16,797	168			
Other umbilical cord/placental abnormalities					
Total	1,293 (7.7)	21 (12.5)	0.02	1.71	1.1 - 2.7
Excessively long umbilical cord	724 (4.3)	11 (6.5)	0.16	-	-
Vasa previa	0 (0)	3 (1.8)	< 0.01	-	-
Single umbilical artery	23 (0.1)	3 (1.2)	< 0.01	13.3	3.9 - 45
Circumvallate placenta	212 (1.2)	5 (3.0)	0.049	2.40	0.98 - 5.9
Succenturiate placenta	112 (0.7)	2 (1.2)	0.41	-	-
Lobed placenta	21 (0.1)	3 (1.8)	< 0.01	14.5	4.3 - 48
Placenta previa	164 (1.0)	5 (3.0)	< 0.01	3.11	1.3 - 7.7
Low lying placenta	105 (0.6)	3 (1.8)	0.06	-	-
Placenta accreta	97 (0.6)	3 (1.8)	0.04	3.13	0.98 - 10

Data are presented as number (%). CI: confidence interval.

Table 3 shows the relation between these umbilical cord/placental abnormalities and the adverse outcomes in the pregnancies complicated by VCI. These umbilical cord/placental abnormalities were not associated with the further adverse outcomes of the pregnancies complicated by VCI.

**Table 3:** Relation Between These Umbilical Cord/Placental Abnormalities and the Adverse Outcomes in the Pregnancies Complicated by Velamentous Cord Insertion

Other umbilical cord/placental abnormalities	P-value
Total	
Preterm delivery	0.24
Fetal asphyxia	0.56
Small-for-gestational-age infant	0.58
Vasa previa	
Preterm delivery	0.23
Fetal asphyxia	0.80
Small-for-gestational-age infant	0.85
Single umbilical artery	
Preterm delivery	0.82
Fetal asphyxia	0.80
Small-for-gestational-age infant	0.85
Lobed placenta	
Preterm delivery	0.82
Fetal asphyxia	0.80
Small-for-gestational-age infant	0.85
Placenta previa	
Preterm delivery	0.51
Fetal asphyxia	0.89
Small-for-gestational-age infant	0.84

## Discussion

VCI is the insertion of the umbilical cord into the membranes of the placenta before reaching the placental margin, and it has been reported to occur in 1.5% of term singleton placentas; however, in pregnancies conceived assisted reproductive technology, the incidence has been observed to rise to 3.65% [12]. This established higher incidence of abnormal cord insertion in IVF conceived pregnancies is more supportive of the trophotropism hypothesis, since the exact chronological succession of biological events, necessary for proper blastocyst implantation, is disturbed at more than one phase in the case of assisted reproduction [1, 12]. In addition, pregnancies conceived through ART have been shown to predispose to low lying placentas and abnormal insertions compared to naturally conceived pregnancies [1, 10], probably due to uterine contractions induced by the embryo placement catheter. Maternal smoking has been also reported to have effect on the local and systemic maternal immune system in decidual tissue and peripheral blood during pregnancy [13]. These conditions support some previous studies [1, 2, 14] concerning the association between the presence of VCI and the adverse perinatal outcomes observed in the current study. In addition, marginal and especially VCI has been observed to be associated with an increased risk of hemorrhage in the third stage of labor, need for manual removal of the placenta and curettage [15]. Therefore, VCI can be identified prenatally and so possibly influence obstetric management.

In the current study, we examined the relation between the presence of other umbilical cord/placental abnormalities and perinatal outcomes in the pregnancies complicated by VCI; however, there were not significant additional influence of the presence of other umbilical cord/placental abnormalities on their adverse outcomes. One of the main reasons leading to the current observations may be the small sample size of this study. In this study, there were only three to five cases of each umbilical cord/placental abnormality. It is estimated that a definitive power to detect the same difference would require at least 12 patients in each group (two-tailed α = 0.05, β = 0.2). One of the other reasons contributing to the current results may be the recent advances in transvaginal ultrasound technics. For example, vasa previa is a velamentous insertion of the umbilical cord in which the blood vessels are present over the cervical os [7, 8]. The cord insertion site usually cannot be determined more frequently in cases of VCI than in the normal cord insertion; however, the diagnosis of vasa previa has been adapted to be easy by transvaginal color Doppler imaging. If these blood vessels rupture during labor, it can have catastrophic effects on the fetus. However, prenatal diagnosis of this condition can allow the safe birth of the newborn and avoid fetal hemorrhage. Fetal asphyxia associated with vasa previa or placenta previa may be able to be avoided by the early diagnosis and careful management of pregnancy and delivery. At last, the perinatal outcomes associated with VCI itself may be too adverse, thus the other umbilical cord/placental abnormalities may not affect the prognosis of the pregnancies complicated by VCI. VCI is a moderate risk condition increasing the risks of prematurity and impaired fetal growth; therefore, a future large research may be needed to focus on the optimal management including the gestational age for delivery of the pregnancies complicated by VCI.

### Conclusion

In conclusions, VCI is associated with an increased risk of adverse perinatal outcomes irrespective of the presence of other umbilical cord/placental abnormalities. Routine identification of the placental cord insertion site should be considered.
